# Association of triglyceride-glucose-body mass index and diabetic retinopathy in Chinese elderly patients with type 2 diabetes: a cross-sectional retrospective study

**DOI:** 10.3389/fendo.2026.1851380

**Published:** 2026-06-17

**Authors:** Yaqin Wei, Ziqing Mai, Mianluan Pan, Xinyan Chen, Jinxin Zheng, Yuhao Lin, Xiaohua Li, Xia Meng, Xinjie Lin, Jianquan Zhang

**Affiliations:** 1 Department of Respiratory and Critical Care Medicine, The Eighth Affiliated Hospital of Sun Yat-Sen University, Shenzhen, Guangdong, China; 2 Department of Endocrinology, The Eighth Affiliated Hospital of Sun Yat-Sen University, Shenzhen, Guangdong, China

**Keywords:** aging, diabetes mellitus, diabetic retinopathy, risk stratification, triglyceride-glucose-body mass index

## Abstract

**Objective:**

This cross-sectional and retrospective study aimed to investigate the relationship between the triglyceride-glucose-body mass index (TyG-BMI) and diabetic retinopathy (DR) in an elderly population with type 2 diabetes (T2D).

**Methods:**

Utilizing data from the Diabetes Complications Early Warning Dataset, we enrolled T2D patients aged >60 years. Multivariate logistic regression and restricted cubic spline (RCS) analyses were applied to assess the TyG-BMI-DR association.

**Results:**

Among 1076 included patients, DR prevalence was 47.68%. After adjusting for covariates, higher TyG-BMI quartiles (Q2-Q4) were significantly associated with increased DR risk compared to Q1, with adjusted odds ratios of 2.058 (95% CI: 1.378 - 3.074, P < 0.001), 1.528 (95% CI: 1.006 - 2.322, P = 0.047), and 2.217 (95% CI: 1.417 - 3.467, P < 0.001), respectively. RCS analysis suggested a nonlinear dose-response association.

**Conclusion:**

TyG-BMI index is positively associated with DR risk in elderly T2D patients and serves as a potential indicator for clinical risk stratification.

## Introduction

Diabetic retinopathy (DR) is one of the most common microvascular complications in patients with diabetes mellitus (DM), and is a major cause of blindness and visual impairment (1). Clinically, as DR worsens, it can cause lasting damage to the retina and loss of vision (2). The global prevalence of DR is currently about 34.6% and is expected to increase to 160.5 million cases by 2045 (3, 4). DR prevention and treatment efforts have focused on controlling blood glucose and glycated hemoglobin (HbA1c) levels in diabetic patients. However, the pathogenesis of DR is a multifactorial process driven by a range of mechanisms and factors, including hypertension, abnormal lipid metabolism, inflammation, and insulin resistance (IR) (5, 6). Abnormal lipid metabolism is a common complication of type 2 diabetes (T2D) and significantly increases the risk of microvascular complications (7). A systematic review indicates that variability in key lipid indicators including LDL, HDL and TG is closely linked to the risk of diabetic microvascular complications (8). Fluctuations in triglyceride and low−density lipoprotein levels may lead to renal microvascular damage including albuminuria and decreased glomerular filtration rate, with neurological injuries also reported (9), highlighting the role of unstable lipid metabolism in the development of diabetic microangiopathy. Earlier DR typically presents with no symptoms. However, if the disease advances to later stages, it can lead to irreversible vision loss and a poor prognosis. Despite ongoing updates in diagnosis and treatment options, their impact remains limited (10). Overall, current screening and diagnosis of DR rely primarily on fundus photography and fluorescein angiography (11, 12). Therefore, identifying biomarkers to assess the DR is essential to optimize clinical treatment and improve patient prognosis.

The triglyceride-glucose (TyG) index is calculated from the fasting triglyceride (TG) and fasting blood glucose (FBG) levels, and the TyG-related parameters, including the TyG index and TyG body mass index (TyG-BMI index), are simple and reliable alternative indicators of insulin resistance. Numerous cross-sectional and retrospective studies have shown that the TyG index and its derivatives are significantly associated with all-cause mortality in critically ill patients suffering from ischemic stroke (13), chronic kidney disease (14), and cardiovascular disease (15, 16). Furthermore, the TyG and TyG-BMI indices, for instance, are strongly linked to disorders of glucose and lipid metabolism, including IR (17, 18), metabolic syndrome (19), and fatty liver disease (20). In addition, the combination of TyG and TyG-BMI index indices can enhance the assessment of hypertension and cardiovascular risk, with TyG−BMI index exhibits high accuracy in identifying individuals with elevated cardiovascular risk (18). HbA1c, blood pressure, renal disease, obesity are associated with T2D and DR. However, the relationship between TyG-BMI index and DR in elderly patients with T2D has not been reported. Therefore, considering its low-cost and data availability, the present study explored the association between TyG-BMI index and the risk of DR, assessed the dose-response relationship of this patient group.

## Materials and methods

### Study population

**Figure 1:**
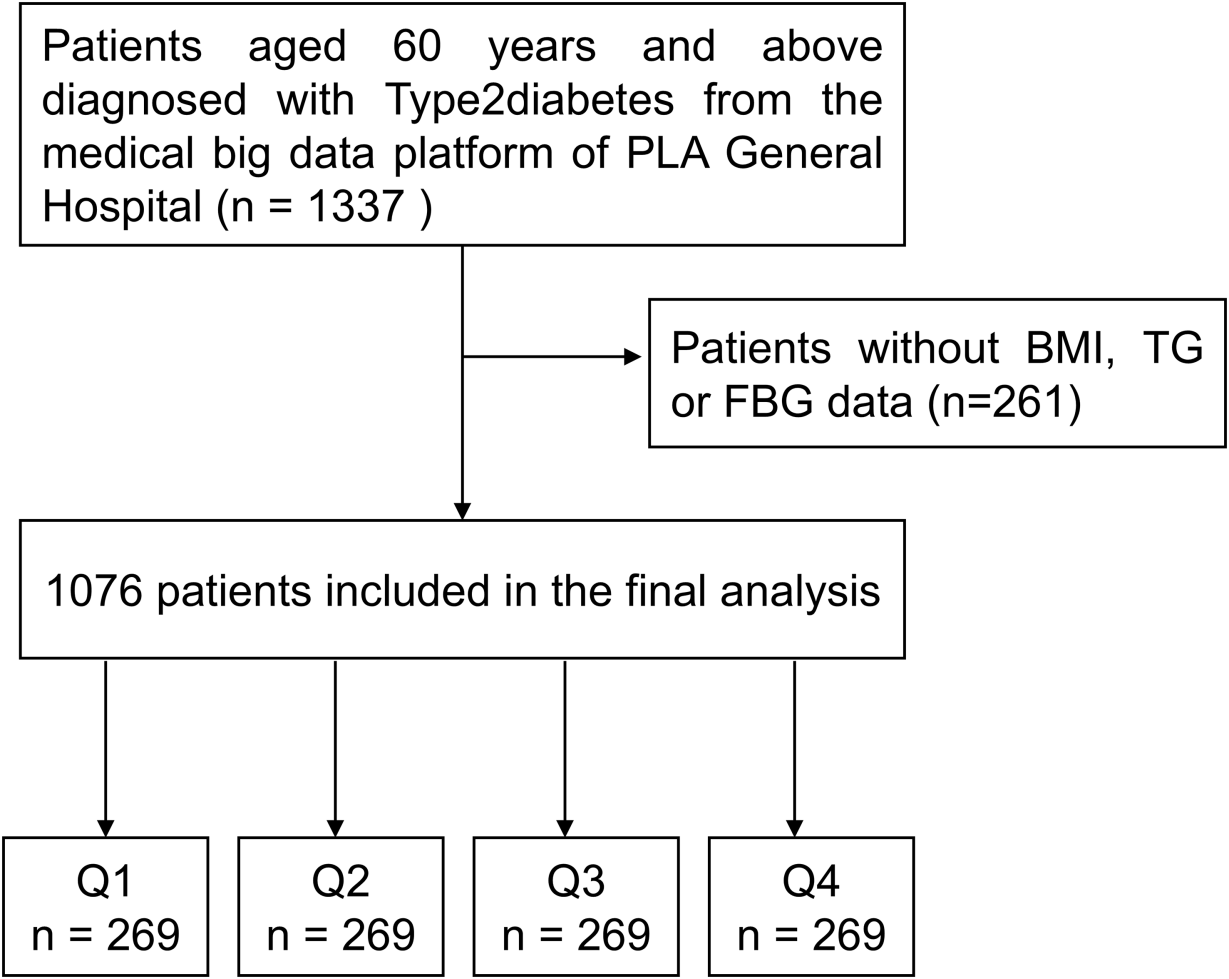
The flow chart.

The data for this study were obtained from the National Population Health Science Data Center Data Warehousing PHDA “Diabetes Complications Early Warning Dataset V1.0” of the 301 Hospital of the People’s Liberation Army (PLA). We obtained and used the data strictly in accordance with the data−use requirements of the platform. All data are derived from inpatient records and pertain to patients who were definitively diagnosed with diabetes and DR by clinicians based on laboratory test results and other findings. This database collects data on diabetes complications from December 2016 to December 2021, documenting the diagnosis of diabetes and DR for patients after their first hospital admission. We included patients over 60 years old with a clear diagnosis of T2D. Patients lacking height, weight, TG and FBG data were excluded. Ultimately, 1,076 participants were finally included in this study after excluding 261 participants due to missing or abnormal data on body weight, height and TG (**Figure 1**).

### Data collection

The TyG index was utilized to assess IR by combining FBG levels with TG measurements. Participants’ weight, height, and laboratory tests for FBG and TG indices were collected to calculate TyG-BMI index. Participants were categorized into four groups (Q1, Q2, Q3, Q4) based on quartiles of TyG-BMI index. The formula for calculating TyG and TyG-BMI index is as follows: TyG = ln [TG (mg/dL) × FBG (mg/dL)/2]; BMI = weight(kg)/height²(m²); TyG-BMI index = TyG × BMI.

Globally recognized diagnostic criteria for diabetes mellitus are based on WHO guidelines (21). The PLA 301 Hospital has confirmed the quality of the data, which was collected, processed, and cleaned by the data provider. The medical instruments used for testing are precise and accurate, hold valid medical device registration certificates, and the testing methods are scientifically sound and reasonable, with standardized operating procedures in place.

Other laboratory indicators include: systolic blood pressure (SBP), diastolic blood pressure (DBP), FBG, Hemoglobin (HB), TG, HbA1c, total cholesterol (TC), high density lipoprotein cholesterol (HDL_C), low density lipoprotein cholesterol (LDL_C), blood urea (BU), blood urea nitrogen (BUN), serum creatinine (SCR), serum uric acid (SUA), packed cell volume (PCV), blood platelet count (PLT), total bilirubin (TBILI), direct bilirubin (DBILI), albumin (ALB), lactate dehydrogenase (LDH-L), alanine transaminase (ALT), aspartate transaminase (AST), gamma-glutamyl transferase (GGT), C-reactive protein (CRP), alkaline phosphatase (ALP), prothrombin time (PT), prothrombin activity (PTA), activated partial thromboplastin time (APTT), indirect bilirubin (IBILI), and globulin (GLO).

### Statistical analysis

Participants were characterized according to quartile groupings of the TyG-BMI index. restricted cubic spline (RCS) analysis was used to assess the assumption of linearity in the relationship between TyG-BMI index and DR events. RCS analysis was performed with four knots placed at the 5th, 35th, 65th, and 95th percentiles of TyG−BMI distribution. To assess multicollinearity among the included variables, the variance inflation factor (VIF) was calculated. Potential covariates were initially selected via univariate logistic regression (P < 0.05) and clinical relevance. To eliminate multicollinearity and achieve dimension reduction, we adopted a VIF threshold of less than 5. Subsequently, LASSO penalized regression was performed to screen core influencing factors for inclusion in the final multivariate regression model. In addition, variables with clinical significance (such as gender and other metabolism-related indicators) were retained. Continuous variables were summarized as median (M) and interquartile range (IQR), and differences between groups were analyzed using the Mann-Whitney U test or the Kruskal-Wallis H test. Categorical variables were expressed as number of cases and percentage (%), and differences between groups were analyzed using the χ2 test. Nonparametric tests were used to compare ordinal variables. Logistic regression was used to adjust for potential confounders, and associations between variable indicators and DRs were expressed as odds ratios (OR) with 95% confidence intervals (CI). Subgroup analyses stratified by hypertension and cardiovascular− cerebrovascular comorbidities were performed on an exploratory basis, given their known roles in DR pathogenesis. Data were analyzed using R software (4.2.2). A two-sided P-value of less than 0.05 indicated a statistically significant difference.

## Results

### Baseline characteristics

This study comprised 1,076 elderly patients with complete TyG-BMI index and clinical data, including 569 males (52.88%) and 507 females (47.12%). The median age was 66 years (interquartile range [IQR]: 63–71 years), and the median TyG-BMI index was 192.23 (IQR: 171.33-214.19). Baseline characteristics of participants with and without DR are presented in **Table 1**. Compared to the non-DR (NDR) group, patients in the DR group exhibited a significantly higher prevalence of hypertension, cerebral apoplexy, carotid artery stenosis, fatty liver disease, biliary tract disease, nephropathy, renal failure, coronary heart disease, respiratory system disease, and arterial disease of diabetes patients (Leaddp). Moreover, the DR group showed elevated levels of SBP, DBP, glucose, TyG, HbA1c, TC, LDL-C, BU, SCR, SUA, LDL-L, and PTA. Importantly, the TyG-BMI index was significantly higher in the DR group than in the NDR group (195.43 vs 189.73, P < 0.001).

**Table 1:** Baseline characteristics of the DR and NDR.

Characteristics	Overall (n=1076)	NDR (n=563)	DR (n=513)	P
Demographic				
Male, n (%)	569 (52.88%)	288 (51.15%)	281 (54.78%)	0.235
Age, year (IQR)	66 (63, 71)	67 (63, 72)	65 (62, 70)	<0.001
BMI, kg/m^2^	25.69 (23.42, 27.98)	25.15 (22.86, 27.51)	25.98 (24.10, 28.35)	<0.001
Laboratory testsc				
SBP, mmHg	140.0 (128.0, 153.8)	137.0 (125.0, 150.0)	140.0 (130.0, 158.0)	<0.001
DBP, mmHg	79.0 (70.0, 85.0)	76.0 (70.0, 84.0)	80.0 (70.0, 86.0)	<0.001
FBG, mmol/L	7.31 (5.73, 9.69)	6.91 (5.71, 8.88)	7.94 (5.77,10.76)	0.001
TG, mmol/L	1.41 (1.03, 2.09)	1.39 (1.01, 2.05)	1.44 (1.04, 2.13)	0.199
TyG index	7.48 (7.02, 7.97)	7.42 (7.00, 7.92)	7.57 (7.08, 8.04)	0.015
TyG-BMI index	192.23 (171.33, 214.19)	189.73 (166.11, 210.89)	195.43 (176.81, 220.03)	<0.001
HbA1c, %	7.50 (6.60, 8.90)	7.10 (6.40, 8.20)	8.20 (7.00, 9.70)	<0.001
TC, mmol/L	4.31 (3.64, 5.18)	4.22 (3.52, 5.06)	4.4 (3.76, 5.28)	0.004
HDL_C, mmol/L	1.06 (0.89, 1.27)	1.06 (0.87, 1.27)	1.07 (0.90, 1.27)	0.139
LDL_C, mmol/L	2.65 (2.08, 3.32)	2.53 (1.97, 3.19)	2.73 (2.20, 3.44)	0.001
Fibrinogen, g/L	3.52 (2.95, 4.41)	3.46 (2.90, 4.30)	3.57 (3.00, 4.50)	0.224
BU, mmol/L	5.92 (4.81, 7.74)	5.54 (4.45, 6.88)	6.47 (5.22, 8.79)	<0.001
SCR, μmol/L	72.75 (59.00, 95.60)	70.10 (58.00, 84.30)	78.60 (60.25, 114.00)	<0.001
SUA, μmol/L	307.70 (251.15, 378.50)	299.60 (241.80, 359.60)	320.10 (257.75, 394.85)	<0.001
HB, g/L	132.00 (118.00, 143.00)	133.00 (121.00, 144.00)	130.00 (116.00, 142.00)	0.002
PCV	0.38 (0.35, 0.42)	0.39 (0.36, 0.42)	0.38 (0.33, 0.41)	<0.001
PLT, ×10^9^	205.00 (169.00, 250.00)	205.00 (170.00, 253.00)	204.00 (168.00, 249.50)	0.345
TBILI, μmol/L	9.50 (6.80, 13.00)	10.00 (7.40, 13.50)	8.90 (6.30, 12.55)	0.001
DBILI, μmol/L	2.80 (1.90, 4.00)	3.10 (2.20, 4.20)	2.50 (1.60, 3.60)	<0.001
TP, g/L	66.30 (62.12, 70.47)	67.20 (63.40, 71.30)	64.90 (61.30, 69.50)	<0.001
ALB, g/L	39.90 (37.00,42.30)	40.50 (37.70, 42.80)	39.50 (35.90, 41.60)	<0.001
LDH_L, U/L	163.10 (142.50, 190.18)	159.70 (140.70, 188.80)	165.00 (145.00, 192.60)	0.430
ALT, U/L	16.40 (11.90, 24.50)	17.60 (12.60, 26.40)	15.30 (11.40, 22.65)	0.003
AST, U/L	16.00 (13.20, 21.00)	16.60 (13.90, 22.40)	15.30 (12.60, 19.80)	<0.001
GGT, U/L	23.05 (16.40, 35.18)	24.50 (17.50, 40.60)	21.20 (15.50, 31.30)	0.004
ALP, U/L	69.20 (57.43, 84.90)	70.40 (58.20, 85.20)	68.40 (56.85, 84.45)	0.094
PT, s	13.10 (12.60, 13.70)	13.20 (12.70, 13.80)	12.90 (12.40, 13.50)	<0.001
PTA, %	98.00 (89.00, 107.00)	97.00 (88.40,106.00)	99.00 (89.00, 108.37)	0.601
APTT, s	35.80 (33.10, 38.70)	35.90 (33.10, 38.90)	35.60 (33.12, 38.20)	0.018
IBILI, μmol/L	6.60 (4.72, 9.30)	6.80 (4.90, 9.50)	6.20 (4.50, 8.85)	0.005
GLO, g/L	26.50 (23.50, 29.70)	27.00 (24.10, 30.00)	25.90 (23.10, 29.20)	0.010
Comorbidities, n (%)				
Hyperlipidemia	237 (22.03%)	142 (25.22%)	95 (18.52%)	0.008
Hypertension	820 (76.21%)	406 (72.11%)	414 (80.70%)	0.001
Atherosclerosis	662 (61.52%)	353 (62.70%)	309 (60.23%)	0.406
Cerebral apoplexy	125 (11.62%)	42 (7.46%)	83 (16.18%)	<0.001
Carotid Artery Stenosis	71 (6.60%)	28 (4.97%)	43 (8.38%)	0.026
Fatty liver disease	267 (24.81%)	95 (16.87%)	172 (33.53%)	<0.001
Cirrhosis	18 (1.67%)	11 (1.95%)	7 (1.36%)	0.454
Chronic liver disease	151 (14.03%)	82 (14.56%)	69 (13.45%)	0.599
Pancreatic disease	17 (1.58%)	8 (1.42%)	9 (1.75%)	0.662
Biliary tract disease	189 (17.57%)	85 (15.10%)	104 (20.27%)	0.026
Nephropathy	467 (43.40%)	151 (26.82%)	316 (61.60%)	<0.001
Renal failure	68 (6.32%)	12 (2.13%)	56 (10.92%)	<0.001
Nervous System Disease	80 (7.43%)	46 (8.17%)	34 (6.63%)	0.336
Coronary Heart Disease	488 (45.35%)	306 (54.35%)	182 (35.48%)	<0.001
Myocardial Infarction	97 (9.01%)	59 (10.48%)	38 (7.41%)	0.080
Congestive Heart Failure	114 (10.59%)	67 (11.90%)	47 (9.16%)	0.146
Arrhythmias	99 (9.20%)	60 (10.66%)	39 (7.60%)	0.085
Respiratory System Disease	205 (19.05%)	104 (18.47%)	101 (19.69%)	0.612
Leaddp	209 (19.42%)	65 (11.55%)	144 (28.07%)	<0.001
Hematonosis	155 (14.41%)	54 (9.59%)	101 (19.69%)	<0.001
Rheumatic Immunity Disease	44 (4.09%)	27 (4.80%)	17 (3.31%)	0.223

BMI, Body mass index; SBP, systolic blood pressure; DBP, diastolic blood pressure; TG, Triglyceride; FBG, fasting blood glucose; HbA1c, hemoglobin a1c; TC, total cholesterol; HDL_C, high density lipoprotein cholesterol; LDL_C, low density lipoprotein cholesterol; BU, blood urea; SCR, serum creatinine; SUA, serum uric acid; HB, hemoglobin; PCV, packed cell volume; PLT, platelets; TBILI, total bilirubin; DBILI, direct bilirubin; TP, total protein; ALB, albumin; LDH_L, lactate dehydrogenase; ALT, alanine aminotransferase; AST, aspartate aminotransferase; GGT, gamma glutamyl transferase; ALP, alkaline phosphatase; PT, prothrombin time; PTA, prothrombin activity; APTT, activated partial thromboplastin time; IBILI, indirect bilirubin; GLO, globulin; Leaddp, Lower Extremity Arterial Disease of Diabetes Patients.

Based on TyG-BMI index quartiles, participants were categorized as follows: Q1 (n=269, TyG-BMI index < 171.33), Q2 (n=269, 171.33 ≤ TyG-BMI index < 192.23), Q3 (n=269, 192.23 ≤ TyG-BMI index < 214.19) and Q4 (n=269, TyG -BMI index ≥ 214.19). Compared with participants in the Q1 quartile, those in higher TyG-BMI quartiles (Q2–Q4) had higher levels of BMI, SBP, DBP, glucose, TG, TyG, HbA1c, TC, LDL-C, FBG, SUA, HB, PCV, TP, ALB, GGT, PTA, APTT, and GLO. Conversely, the Q4 group demonstrated lower levels of HDL-C, TBILI, DBILI, and PT relative to Q1. Moreover, patients in the highest TyG−BMI quartile exhibited a higher DR risk, hyperlipidemia, hypertension, atherosclerosis, stroke, cirrhosis, nephropathy, and Leaddp, as summarized in **Table 2**.

**Table 2:** Baseline characteristics according to TyG-BMI index quartiles.

Characteristics	Q1 (< 171.33)	Q2 (171.33-192.23)	Q3 (192.23-214.19)	Q4 (≥214.19)	P
Demographic					
Male, n (%)	130 (48.33%)	142 (52.79%)	156 (57.99%)	141 (52.42%)	0.166
Age, year (IQR)	68 (64, 73)	66 (63, 70)	65 (62, 70)	65 (62, 71)	<0.001
BMI, kg/m^2^	22.33 (20.70, 23.60)	24.96 (23.6, 26.22)	26.56 (25.2, 27.75)	29.37 (27.66, 31.20)	<0.001
Laboratory tests, IQR					
SBP, mmHg	138.0 (126.5, 150.0)	140.0 (126.5, 153.5)	140.0 (128, 155)	140.0 (130.0, 158.0)	0.037
DBP, mmHg	75.0 (68.0, 80.0)	80.0 (70.0, 85.0)	80.0 (72.0, 88.0)	80.0 (70.0, 86.5)	<0.001
FBG, mmol/L	6.13 (5.00, 7.76)	6.75 (5.67, 9.10)	7.45 (6.05, 10.14)	9.02 (7.25, 12.64)	<0.001
TyG index	6.90 (6.51, 7.29)	7.33 (7.00, 7.70)	7.68 (7.29, 8.05)	8.04 (7.71, 8.60)	<0.001
TyG-BMI index	156.81 (143.22, 165.02)	182.75 (177.13, 187.71)	202.74 (197.63, 207.49)	233.55 (222.48, 251.02)	<0.001
HbA1c, %	7.00 (6.30, 8.15)	7.30 (6.60, 8.80)	7.60 (6.60, 8.80)	8.20 (7.00, 9.70)	<0.001
TC, mmol/L	4.05 (3.43, 4.75)	4.21 (3.57, 4.98)	4.28 (3.62, 5.19)	4.74 (4.06, 5.63)	<0.001
HDL_C, mmol/L	1.19 (0.98, 1.44)	1.07 (0.90, 1.30)	1.00 (0.86, 1.16)	1.00 (0.84, 1.18)	<0.001
LDL_C, mmol/L	2.41 (1.88, 3.05)	2.63 (2.08, 3.18)	2.68 (2.07, 3.35)	2.84 (2.29, 3.58)	<0.001
Fibrinogen, g/L	3.51 (2.94, 4.46)	3.43 (2.90, 4.27)	3.49 (2.95, 4.16)	3.78 (3.00, 4.77)	0.029
BU, mmol/L	5.79 (4.58, 7.97)	5.81 (4.81, 7.24)	6.03 (4.86, 7.78)	6.01 (4.95, 7.92)	0.521
SCR, μmol/L	70.40 (57.15, 96.05)	71.80 (58.65, 91.50)	74.80 (59.40, 95.75)	75.20 (60.20, 97.4)	0.518
SUA, μmol/L	291.70 (231.75, 367.00)	292.60 (239.40, 352.45)	317.00 (256.30, 398.20)	328.90 (274.90, 391.35)	<0.001
HB, g/L	127.00 (111.00, 138.00)	132.00 (119.00, 142.00)	135.00 (121.00, 145.50)	133.00 (120.00, 146.31)	<0.001
PCV	0.37 (0.33, 0.40)	0.39 (0.35, 0.42)	0.39 (0.36, 0.42)	0.39 (0.35, 0.42)	<0.001
PLT, ×10^9^	203.00 (167.50, 251.00)	203.00 (165.00, 252.50)	206.00 (168.00, 254.50)	208.00 (176.00, 247.50)	0.74
TBILI, μmol/L	9.10 (6.8, 13.04)	10.00 (7.05, 13.75)	9.80 (7.05, 12.9)	8.80 (6.40, 12.25)	0.044
DBILI, μmol/L	2.80 (1.9, 4.05)	3.00 (2.05, 4.40)	2.90 (2.00, 3.98)	2.50 (1.65, 3.60)	0.004
TP, g/L	65.50 (61.55, 69.55)	65.60 (62.00, 69.70)	67.30 (62.50, 71.35)	66.90 (62.4, 71.50)	0.014
ALB, g/L	39.40 (35.85, 41.85)	40.00 (37.60, 42.10)	40.70 (37.80, 43.05)	39.70 (36.65, 42.40)	0.002
LDH_L, U/L	160.70 (140.90, 192.30)	160.70 (142.50, 186.20)	162.50 (142. 40, 188.00)	166.90 (146.20, 197.10)	0.281
ALT, U/L	15.80 (10.95, 26.65)	16.50 (11.85, 22.6)	16.30 (12.65, 25.55)	17.40 (12.20, 24.95)	0.329
AST, U/L	16.20 (13.20, 22.05)	15.60 (13.15, 19.50)	16.10 (13.50, 21.40)	16.20 (12.65, 21.30)	0.421
GGT, U/L	20.20 (14.80, 32.60)	22.00 (15.80, 33.65)	23.80 (17.65, 37.05)	25.90 (18.10, 38.35)	<0.001
ALP, U/L	70.50 (57.00, 85.50)	68.10 (55.75, 82.30)	69.00 (58.05, 86.15)	70.10 (58.80, 85.45)	0.465
PT, s	13.20 (12.70, 13.80)	13.00 (12.60, 13.60)	13.10 (12.60, 13.80)	12.90 (12.30, 13.45)	<0.001
PTA, %	96.00 (86.00, 104.00)	99.00 (90.00, 107.00)	97.10 (89.00, 108.00)	100.00 (90.80, 110.00)	0.001
APTT, s	36.20 (33.70, 39.00)	35.80 (33.15, 39.00)	35.70 (32.85, 38.20)	35.30 (32.55, 37.95)	0.025
IBILI, μ mol/L	6.30 (4.60, 8.75)	7.20 (4.90, 9.85)	6.70 (4.90, 9.20)	6.30 (4.40, 8.70)	0.104
GLO, g/L	26.30 (23.45, 29.40)	25.90 (23.20, 28.65)	26.6 (23.40, 29.20)	27.5 (24.25, 30.90)	0.006
Comorbidities, n (%)					
DR	99 (36.80%)	137 (50.93%)	129 (47.96%)	148 (55.02%)	<0.001
Hyperlipidemia	42 (15.61%)	57 (21.19%)	67 (24.91%)	71 (26.39%)	0.013
Hypertension	187 (69.52%)	202 (75.09%)	213 (79.18%)	218 (81.04%)	0.009
Atherosclerosis	151 (56.13%)	158 (58.74%)	173 (64.31%)	180 (66.91%)	0.039
Cerebral apoplexy	31 (11.52%)	16 (5.95%)	43 (15.99%)	35 (13.01%)	0.003
Carotid Artery Stenosis	11 (4.09%)	17 (6.32%)	17 (6.32%)	26 (9.67%)	0.075
Fatty liver disease	18 (6.69%)	70 (26.02%)	89 (33.09%)	90 (33.46%)	<0.001
Cirrhosis	6 (2.23%)	5 (1.86%)	7 (2.60%)	0 (0.00%)	0.088
Chronic liver disease	44 (16.36%)	38 (14.13%)	43 (15.99%)	26 (9.67%)	0.098
Pancreatic disease	7 (2.60%)	2 (0.74%)	2 (0.74%)	6 (2.23%)	0.175
Biliary tract disease	43 (15.99%)	48 (17.84%)	52 (19.33%)	46 (17.10%)	0.778
Nephropathy	102 (37.92%)	103 (38.29%)	124 (46.10%)	138 (51.30%)	0.003
Renal failure	17 (6.32%)	20 (7.43%)	14 (5.20%)	17 (6.32%)	0.770
Nervous System Disease	19 (7.06%)	17 (6.32%)	24 (8.92%)	20 (7.43%)	0.705
Coronary Heart Disease	117 (43.49%)	113 (42.01%)	122 (45.35%)	136 (50.56%)	0.210
Myocardial Infarction	31 (11.52%)	17 (6.32%)	20 (7.43%)	29 (10.78%)	0.099
Congestive Heart Failure	30 (11.15%)	21 (7.81%)	27 (10.04%)	36 (13.38%)	0.205
Arrhythmias	27 (10.04%)	20 (7.43%)	26 (9.67%)	26 (9.67%)	0.713
Respiratory System Disease	56 (20.82%)	47 (17.47%)	56 (20.82%)	46 (17.10%)	0.535
Leaddp	38 (14.13%)	59 (21.93%)	50 (18.59%)	62 (23.05%)	0.041
Hematonosis	44 (16.36%)	41 (15.24%)	32 (11.90%)	38 (14.13%)	0.499
Rheumatic Immunity Disease	18 (6.69%)	7 (2.60%)	11 (4.09%)	8 (2.97%)	0.072

### Correlation analysis of TyG-BMI index and DR

Logistic regression models with different adjustment strategies were used to assess the association between TyG-BMI and DR.In the crude unadjusted model (Model 1), each ten-unit increment in continuous TyG-BMI was significantly linked to higher DR risk (OR = 1.259, 95%CI: 1.129 – 1.430, P < 0.001). After adjustment for age and sex (Model 2), the association remained stable (OR = 1.233, 95%CI: 1.105 – 1.376). Further adjustment for multiple clinical and laboratory covariates in the fully adjusted model (Model 3) still yielded a significant positive association (OR = 1.224, 95%CI: 1.063 – 1.410, P = 0.005).

**Table 3** presents the association between TyG-BMI index and DR. When TyG-BMI was categorized into four quartiles with Q1 (<171.33) as the reference group, the crude ORs of DR for Q2, Q3 and Q4 were 2.018 (95%CI: 1.427 – 2.852, P < 0.001), 1.614 (95%CI: 1.142 – 2.281, P = 0.007) and 2.344 (95%CI: 1.656 – 3.317, P < 0.001), respectively. These statistical significances persisted across age-sex adjusted and fully adjusted models. Notably, the quartile-based risk distribution did not show a strict monotonic upward trend, which supported a non-linear association pattern.

**Table 3:** Association between TyG-BMI index and DR.

TyG-BMI index quartile	Model-1		Model-2		Model-3	
	OR (95% CI)	P value	OR (95% CI)	P value	OR (95% CI)	P value
TyG-BMI index^a^						
Continues variable	1.259 (1.129, 1.430)	<0.001	1.233 (1.105, 1.376)	<0.001	1.224 (1.063, 1.410)	0.005
Q1	ref	–	ref	–	ref	–
Q2	2.018 (1.427, 2.852)	<0.001	1.876 (1.322, 2.663)	<0.001	2.058 (1.378, 3.074)	<0.001
Q3	1.614 (1.142, 2.281)	0.007	1.490 (1.049, 2.118)	0.025	1.528 (1.006, 2.322)	0.047
Q4	2.344 (1.656, 3.317)	<0.001	2.201 (1.550, 3.125)	<0.001	2.217 (1.417, 3.467)	<0.001

Model-1: No adjustment is made.

Model-2: Adjusted for age, sex.

Model-3: Adjusted for age, sex, HbA1c, LDL_C, BU, PCV, DBILI, ALB, SCR, AST, GGT, hypertension, renal failure, cerebral apoplexy.

^a^TyG-BMI index: Q1: TyG-BMI index < 171.33; Q2:171.33 ≤ TyG-BMI index < 192.23; Q3:192.23 ≤ TyG-BMI index < 214.19; Q4: TyG-BMI index ≥ 214.19. Correlation analysis of TyG-BMI index and DR.

**Figure 2:**
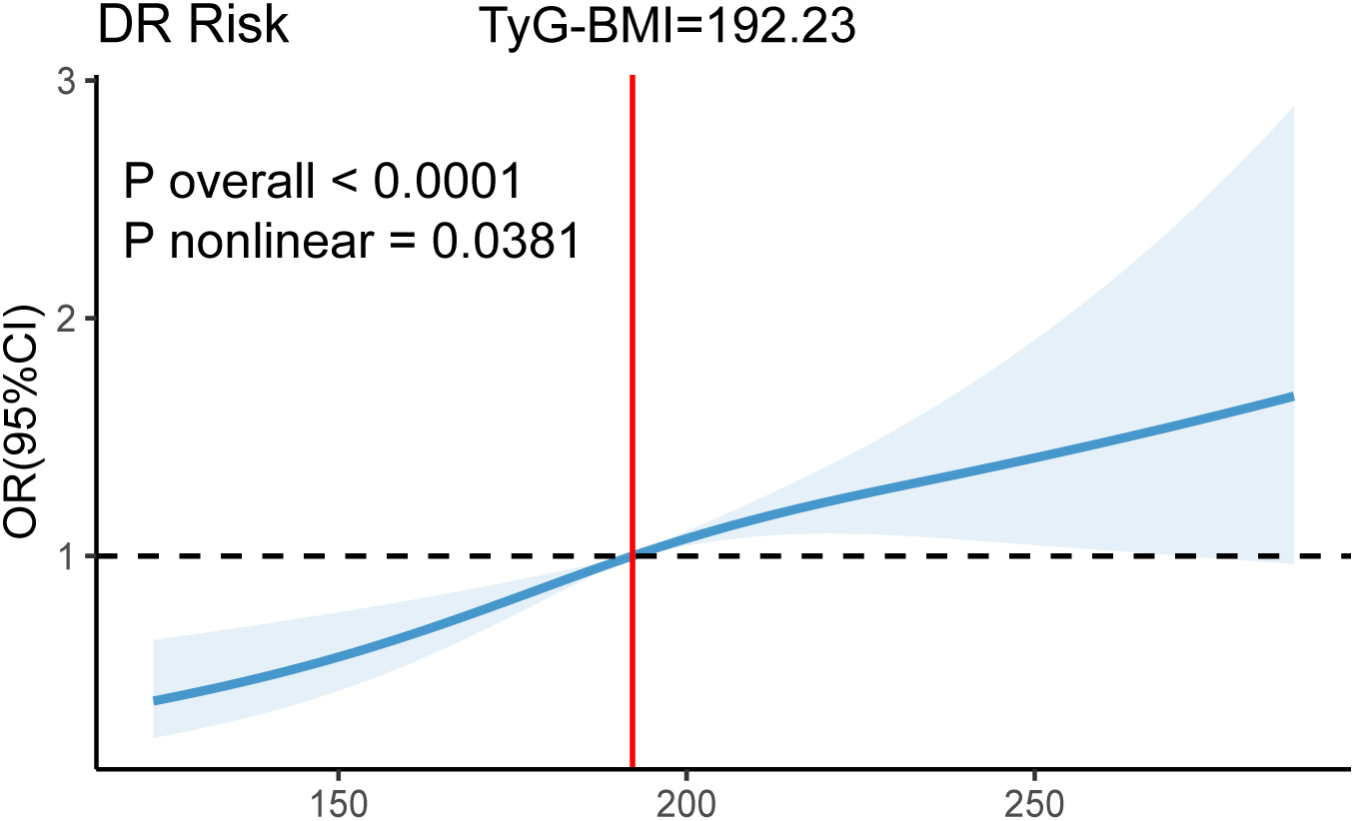
RCS analysis of the TyG-BMI index for the risk of DR.

To explore the dose−response relationship between the TyG− BMI index and DR risk using RCS regression. The adjusted RCS curve displayed an overall positive association with a nonlinear trend, and non−linearity testing confirmed a statistically significant nonlinear relationship (non-linearity test, P = 0.0381) (**Figure 2**).

### Subgroup analysis

**Figure 3:**
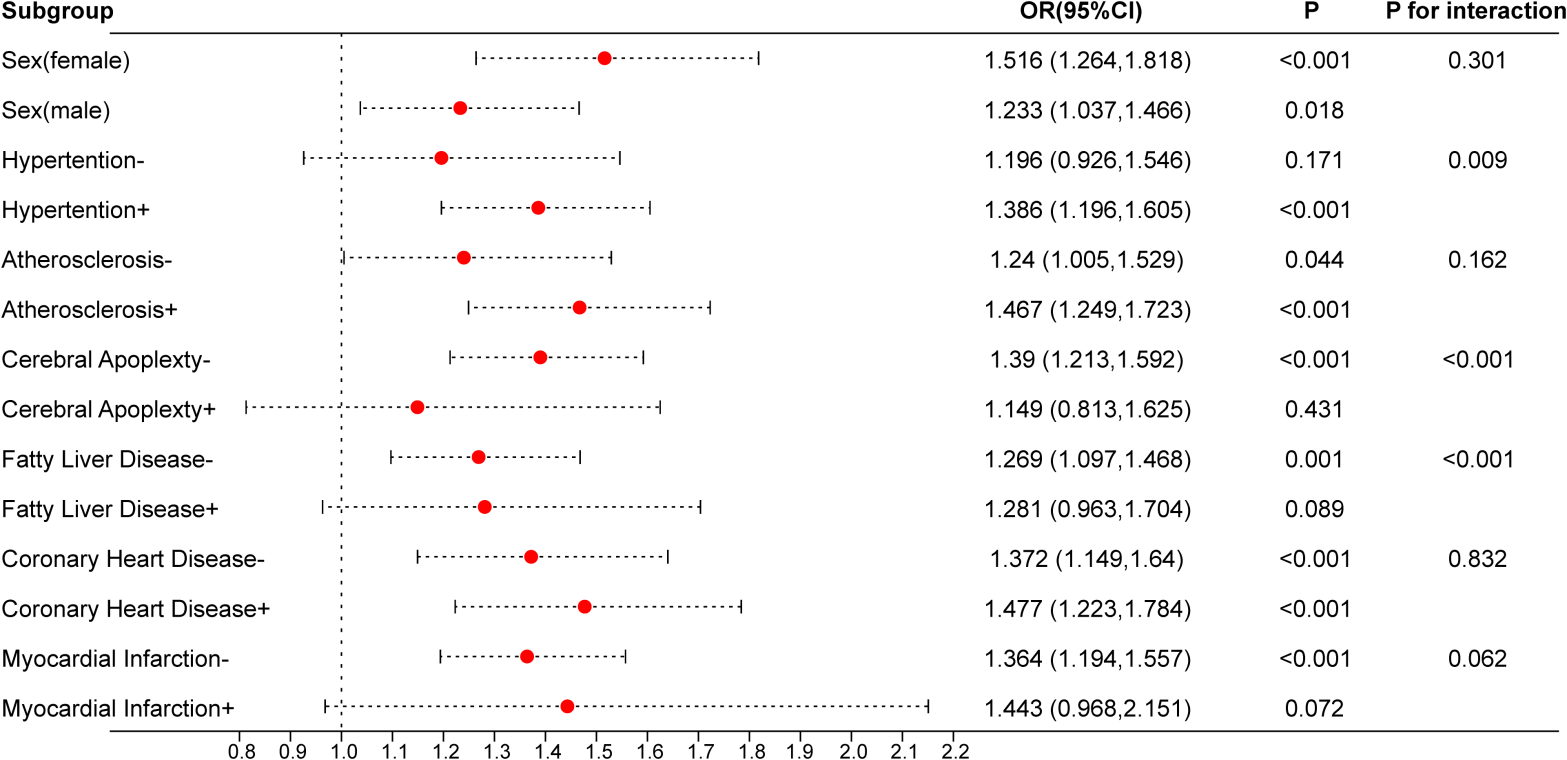
Forest plot of multivariate logistic regression subgroup analysis for the associations of TyG-BMI index and DR.

To explore potential effect modifiers for the TyG-BMI-DR association, we conducted stratified analyses according to sex, hypertension, atherosclerosis, atherosclerosis, cerebral apoplexy, coronary heart disease, fatty liver disease, and myocardial infarction (**Figure 3**). In the fully adjusted regression model, the positive correlation between TyG-BMI and DR was stronger among patients with hypertension, with a corresponding OR of 1.386 (95%CI: 1.196–1.605, P < 0.001). Interaction analyses confirmed significant effect modification by hypertension (P for interaction = 0.009), cerebral apoplexy and fatty liver disease (all P < 0.001), suggesting that the magnitude of the association between TyG-BMI index and DR risk differed substantially across these clinical subgroups.

## Discussion

Diabetes is particularly highly prevalent among older populations, with a global prevalence of 24.4% in those aged over 75 years, who face an elevated risk of severe diabetic complications due to increased medical complexity and frailty (22). The aim of this study was to assess the relationship between the TyG−BMI index and prevalent DR among elderly patients with T2D. Our findings demonstrated a significant positive association between higher TyG−BMI levels and increased odds of prevalent DR. In addition, RCS analysis confirmed this positive correlation, which remained stable after adjustment for potential confounders. Quartile−based stratified analyses revealed an overall positive association, whereas OR values exhibited local fluctuations across subgroups, further supporting a nonlinear dose−response relationship rather than a strictly linear trend between TyG−BMI and DR. Collectively, these results suggest that TyG−BMI may represent an independent correlate of prevalent DR in elderly patients, and may provide supplementary information for clinical maker assessment in patients complicated by renal failure, hypertension, and other cardiovascular diseases.

Several studies have indicated that combining the TyG index with obesity−related indicators such as BMI improves the assessment of IR (23). Compared with single lipid parameters, lipid ratios, glycemic indices, and other obesity−related markers, the TyG−BMI index has shown greater diagnostic performance for IR, reflected by higher AUC values (24). However, the association between BMI and DR remains inconsistent across previous studies. For instance, the Shanghai Diabetic Complications Study identified obesity as a risk factor for DR (25), whereas data from the Singapore Epidemiology of Eye Diseases cohort suggested an inverse correlation between BMI and DR presence and severity (26). Increasing findings have also been reported in Western populations: the Dutch Hoorn Study observed a positive association between higher BMI and incident diabetes (27), while Man et al. reported lower DR lesion incidence with increased BMI in Asian patients with T2D (28). Insulin resistance also plays a pivotal role in DR pathogenesis; Rask− Madsen et al. highlighted that IR exacerbates retinal microvascular injury, contributing to the development of DR (29).

As a composite marker integrating the TyG index and BMI, TyG−BMI provides a more comprehensive evaluation of metabolic dysfunction than the TyG index alone. Incorporating BMI helps reflect the contribution of obesity to IR and diabetic microvascular complications including DR. Insulin resistance is central to DR pathogenesis by inducing microvascular damage and promoting DR onset and progression (30, 31). Moreover, active visceral adipose tissue may secrete pro−inflammatory cytokines such as TNF−α and IL−6, which damage retinal micro vessels (32). By estimating body fat status via BMI, TyG−BMI enables integrated assessment of metabolic and anthropometric correlates of DR risk. The combined effects of insulin resistance and obesity further increase oxidative stress and impair endothelial function, both key drivers of DR development (33). Therefore, TyG−BMI may offer more comprehensive insights into DR pathophysiology than individual markers such as BMI or TyG index.

The present findings also highlight the potential importance of obesity management for mitigating diabetic complications. Central obesity is a modifiable factor closely linked to IR and DR. Although this cross−sectional analysis cannot establish causal effects, existing evidence implies that lifestyle interventions including dietary modification and physical activity may help reduce DR−related risk among individuals with elevated TyG−BMI (34). DR is often asymptomatic at early stages. Given that BMI, triglycerides, and fasting blood glucose are routinely measured in clinical practice, TyG−BMI is a simple and low−cost marker for evaluating prevalent DR−related risk. Nevertheless, its utility as an early screening or predictive tool for incident DR remains unvalidated and requires further prospective verification. Combining easily accessible clinical indicators, TyG−BMI may help identify older diabetic patients with higher prevalent DR risk, who may warrant enhanced clinical attention and regular surveillance, including optimized glycemic control adjustment (35). In addition, integrating TyG−BMI with established markers such as HbA1c may improve comprehensive metabolic assessment. For example, patients with well−controlled HbA1c but high TyG−BMI may still carry higher DR risk driven by underlying obesity and IR, emphasizing the necessity of multi− dimensional evaluation of metabolic dysfunction for diabetic complication prevention (36).

Our subgroup analysis indicated that the TyG−BMI index was associated with the presence of DR among patients with hypertension (OR 1.386, 95% CI 1.196-1.605). This finding is consistent with previous studies emphasizing the role of systemic factors, such as blood pressure, in the progression of DR. Hypertension exacerbates retinal vascular stress, which may increase retinal capillary permeability and promote the presence of DR. These common mechanisms suggest that patients with hypertension and DR are particularly susceptible to microvascular complications and may benefit from targeted interventions against IR and metabolic dysregulation. In patients with diabetes without comorbidities such as cerebrovascular accident (OR 1.390, 95% CI 1.213-1.592) and fatty liver (OR 1.269, 95% CI 1.097-1.468), the TyG-BMI index remained associated with DR. These findings emphasize the importance of careful monitoring and timely assessment of other diagnostic indicators in these patient groups to prevent adverse outcomes. Notably, the mean age of patients in the DR group was lower than in the NDR group. It seems the correlation between TyG-BMI index and DR was pronounced in the lower age subgroups of the elderly, a finding that has been reported in a previous study (29), challenging the general clinical interest in older patients, who are often perceived to have a higher comorbidity burden and therefore receive more attention.

While our findings offer preliminary evidence for the association between TyG−BMI and DR, several limitations of this study must be acknowledged. First, this was a single−center retrospective study, which may introduce unavoidable selection bias and limit generalizability to other populations. The biological and clinical relevance of TyG-BMI may differ between mild non-proliferative disease, severe non-proliferative disease, proliferative retinopathy, and diabetic macular edema. Second, detailed information regarding hypoglycemic and other anti−diabetic treatments among inpatients was unavailable, which may affect the predictive reliability of the TyG−BMI index. In addition, we lacked data on diabetes duration, antihypertensive and lipid-lowering therapy, smoking, alcohol consumption, physical activity and ophthalmologic screening access, potentially resulting in residual confounding. Third, only hospitalized patients aged over 60 years were included; therefore, our results may not be applicable to other age groups. Notably, the relatively high DR prevalence (47.68%) in the present study indicated that our participants were predominantly hospitalized elderly diabetic patients with relatively multiple comorbidities, which differed from general community-dwelling type 2 diabetic populations. Fourth, subgroup and interaction analyses were post−hoc exploratory investigations without pre−specified hypotheses or adjustment for multiple testing, which may lead to potential false−positive findings. Accordingly, the conclusions derived from these subgroup analyses should be interpreted cautiously and require validation in larger prospective studies with predefined designs. Fifth, data on the severity grades of DR were not available in our cohort. Given that the biological links between metabolic dysfunction and mild, moderate, or proliferative DR may differ, we were unable to further analyze the dose-response relationship between TyG-BMI and the severity of DR.

## Conclusion

Higher TyG−BMI index is associated with prevalent DR in elderly patients and serves as a potential indicator for clinical risk stratification. Nevertheless, this study fails to establish causality, solid predictive efficacy, screening potential and definite clinical applicability. Further prospective longitudinal studies are needed to determine its predictive relevance for incident DR and clinical screening utility.

## Data Availability

The original contributions presented in the study are included in the article/supplementary material. Further inquiries can be directed to the corresponding author/s.
